# Some Cases of Drug Eruptions

**Published:** 1898-04

**Authors:** W. H. Hudson

**Affiliations:** LaFayette, Ala.


### SOME CASES OF DRUG ERUPTIONS.

BY W. H. HUDSON, M.D.,
LAFAYETTE, ALA.

In 1889 Mrs. T., white, aged thirty years, was under my care for the treatment of a cough which had remained from an attack of pleurisy. Sulphate of codeine was given in one-half grain quantities. After the third dose was taken she sent for me in great alarm on account of an eruption which implicated most of the body. Itching was very intense, and in many places large wheals had developed, presenting the appearance of usual urticaria. The codeine was stopped and the eruption disappeared in a short time.

During the autumn of 1887 we had an epidemic of typhoid fever, most of the cases beginning with the characteristic symptoms. Mr. A., aged nineteen years, was taken sick with chill and high fever. I saw him a few hours after the onset of his sickness. This was before my day of examining the blood for the malarial organism, and I gave him twenty grains of quinine in two doses. A severe urticaria developed, which he informed me came from the quinine, which he said always produced such an eruption on him. The quinine was stopped and the eruption disappeared promptly. The case developed into typical typhoid fever which lasted about a month. This was, perhaps, a case of typhoid of sudden onset. Since that time I have seen many cases of typhoid which began suddenly. Some of them being differentiated from appendicitis with difficulty.

For some years a prominent Alabamian consulted me frequently for minor ailments. During the spring of 1893 he was returning home after an absence of several days, and on his way to his residence from the train stopped in my office to get a simple tonic, as he said he felt run down. I gave him a prescription for the elixir of calisaya. This was on Friday afternoon. On Sunday morning I met him on his way to church; he said he felt all right, but said to me: "Why did you give me that quinine?" I answered that I did not give him quinine, forgetting for a moment that the calisaya contained quinine. He replied: "I know you have given me quinine, for it has done me as quinine always does." Then I remembered the effect that quinine had on him, for he had frequently told me of his idiosyncrasy towards quinine.

His brother, also a prominent Alabamian, was affected the same way by quinine. Some hours after taking the smallest dose of quinine, two or three grains or less, an itching would begin on the entire surface of the glans penis after a few hours an intense redness would appear, then after about ten hours the epithelium of the glans would begin to exfoliate; this would leave a raw and painful surface which would last two or three days before the epithelium would begin to be restored. Quinine affected him in no other way peculiarly. Latterly this gentleman has found that the addition of gum camphor to the quinine prevents the peculiar effect.

In the spring of 1897, Mr. A., aged seventy-eight years, consulted me for a difficulty in passing his urine. He was suffering from an enlarged prostate gland; his urine was purulent and highly ammoniacal. With the exception of the bladder trouble he was a man wonderfully well preserved for one of his age. His arteries, heart, brain, and everything except his prostate gland were much younger than is usually seen in men of even seventy years. I was never able to catheterize his bladder, so I could not wash his bladder out. Among other things which I did for him, I gave boracic acid in 10-grain and salol in 5-grain doses combined in one powder three times a day. He improved rapidly, the urine soon becoming clear, and he regained much control of his bladder. After a few days I reduced the boracic acid to 5 grains and the salol to $2\frac{1}{2}$ grains to the powder. His bladder continued to get better, and I did not see him again for about a month. At this time I was sent for on account of the strange condition he was in. His face was swollen and somewhat indurated, and of a dull, red color; his upper lids were so much swollen that he could open his eyes only with difficulty; there was an intense erythema over his entire body, the itching was so severe that he could not sleep, and the skin everywhere—more marked on his body—was swollen, making the appearance of a most exaggerated scarlet fever. There were a few superficial sores on his hands and arms and on his feet and legs.

Some of these sores were weeks in healing. I suspended the boracic acid and salol, and very soon the symptoms began to ameliorate, the redness and swelling in the skin disappeared, and after a few days such desquamation began, the like of which I had never seen before. The skin of the soles of the feet and palms of the hands came off in great strips, leaving very sensitive, delicate skin underneath. Several of the finger- and toe-nails were also shed. With the exception of the ulcers on the arms, hands and legs he rapidly recovered from the effects of the drugs, but his urine soon became clouded again, and he insisted on having more of the powders. I gave him a powder containing 2 grains of boracic acid and 1 grain of salol, watching the time for the ill effects should they recur. In about three days his hands began to itch, his upper eyelids to itch and swell, and his entire skin to feel a little unnatural—the old trouble was beginning again, although the medicines were given in such small quantities. I stopped the drugs and the symptoms disappeared entirely in a few days. This patient was hardly willing for a radical surgical procedure so long as he was in such good condition, and is still, without further treatment, in fair health.

In another case of a woman with cystitis, I gave boracic acid and salol in the usual quantities; an eruption, probably of a bullous nature, was produced, but the cystitis was greatly improved. This woman lived some distance from me, and I was not informed of the eruption until the medicine had been all taken. Her husband came to see me for further treatment and told me of the condition of her skin. He thought it was the disease "coming out" and was very much pleased. I had the woman brought to me, and much of her body was covered with small healing sores. She had had no medicine for some days and the skin was rapidly returning to a natural condition.

The eruption in these cases was no doubt due to the boracic acid and not to the salol, but what surprised me was the very small quantities of the drug which produced the disturbance in the skin of the first case, and the very severe eruption in the second case, which was produced by the moderate doses.

Frequently, previous to my experience with boracic acid in these cases, I had used this drug as a harmless one, giving it internally in large doses, using it in water as an injection in the bowels of infants, and in many other ways. But it appears that harm may come of it, and we should use it knowing that it is not an entirely safe drug in all cases.

